# Patient characteristics and burden of disease in Japanese patients with generalized pustular psoriasis: Results from the Medical Data Vision claims database

**DOI:** 10.1111/1346-8138.16022

**Published:** 2021-07-01

**Authors:** Akimichi Morita, Nirali Kotowsky, Ran Gao, Reiko Shimizu, Yukari Okubo

**Affiliations:** ^1^ Department of Geriatric and Environmental Dermatology Nagoya City University Graduate School of Medical Sciences Nagoya Japan; ^2^ Boehringer Ingelheim Pharmaceuticals Inc. Ridgefield CT USA; ^3^ Nippon Boehringer Ingelheim Co., Ltd. Tokyo Japan; ^4^ Department of Dermatology Tokyo Medical University Hospital Tokyo Japan

**Keywords:** generalized pustular psoriasis, psoriasis, pustular psoriasis, rare, real‐world evidence

## Abstract

Generalized pustular psoriasis (GPP) is a rare and severe systemic, neutrophilic skin disease. To date, accurate clinical profiling of patients with GPP remains poorly understood. In this study, we present the characteristics and estimate the burden of disease in patients with GPP compared with those with plaque psoriasis, in Japan. This retrospective study was conducted using the Medical Data Vision database between January 1, 2015, and December 31, 2019. Patients with at least one confirmed inpatient or outpatient diagnostic code for GPP (L40.1) or psoriasis vulgaris (L40.0) were included for analysis. The main outcome measures included comparisons of the prevalence of comorbidities, medication use, and healthcare resource utilization between patients with GPP, patients with plaque psoriasis, and a general population‐matched cohort. In total, 718 patients with GPP and 27,773 patients with plaque psoriasis were identified. Patients with GPP were more likely to be female than those with plaque psoriasis (51.6% vs. 38.7%). During the 12‐month follow‐up period, patients with GPP were more likely to experience comorbidities than those with plaque psoriasis, including psoriatic arthritis, other forms of psoriasis, osteoporosis, interstitial pneumonia, and peptic ulcer disease. Medication use also differed between those with GPP and those with plaque psoriasis: patients with GPP were more likely to be prescribed antibiotics and psychiatric medication. Patients with GPP were also more likely to require more healthcare resource utilization with longer hospitalizations than those with plaque psoriasis. Overall, in Japan, patients with GPP have a higher burden of illness than those with plaque psoriasis.

## 1 INTRODUCTION

Generalized pustular psoriasis (GPP) is a rare, severe, neutrophilic skin disease characterized by episodes of widespread eruption of sterile, visible pustules, which can occur with or without systemic inflammation and with or without plaque psoriasis.^1^, ^2^ GPP is associated with potentially life‐threatening systemic complications including sepsis and cardiac failure, and is described by the Japan Ministry of Health as an intractable disease.^1^, ^2^, ^3^, ^4^

Mutations in *IL36RN*, *CARD14*, or *AP1S3* have been found in patients with GPP.^5^, ^6^ Small Japanese studies have identified a number of different *IL36RN* mutations in patients with GPP.^7^, ^8^, ^9^ Patients with these mutations may be at risk of earlier onset of disease and more severe disease.^10^ Indeed, one small study (*n* = 31 patients with GPP) has reported that Japanese patients with GPP alone (*n* = 11) have a higher prevalence of mutations in *IL36RN* than those with concomitant plaque psoriasis (*n* = 20).^11^ Compared with the frequency of *IL36RN* mutations reported in patients with GPP in other regions of the world, the frequency in Japanese patients with GPP (45.2%) is higher than in other East and South Asian populations (28.8% and 0.0%, respectively) and European populations (29.4─34.7%).^12^, ^13^, ^14^ Also, an analysis in 30 Japanese patients with GPP identified a *CARD14* variant as a predisposing factor for GPP with plaque psoriasis, but not in patients with plaque psoriasis only.^15^ This suggests that the pattern of disease may be different in Japanese patients than in many other populations around the world. As such, the clinical profile of Japanese patients with GPP warrants further investigation.

In Japan, treatment guidelines for patients with GPP, as well as criteria for the diagnosis and severity of GPP, were revised in 2014.^2^ Unlike in the USA or Europe, where no treatments are approved for the treatment of GPP specifically, there are currently eight approved biologics for the treatment of GPP in Japan. These are adalimumab, infliximab, certolizumab pegol, risankizumab, guselkumab, secukinumab, brodalumab, and ixekizumab.^2^, ^16^

Published data on the clinical characteristics of Japanese patients with GPP are sparse. This retrospective study using the Japanese Medical Data Vision (MDV) database was conducted to characterize the clinical profile of patients with GPP and assess the burden of disease, including healthcare resource utilization (HCRU), compared with those with plaque psoriasis. Outcome measures included comorbidities, dermatologic medication use, concomitant medication use, and all‐cause HCRU during the 12‐month follow‐up data collection period.

## 2 METHODS

**FIGURE 1:**
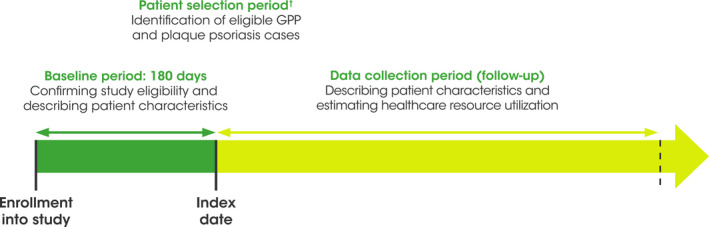
Study design. ^†^Patients were eligible to enter a cohort when they fulfilled all study criteria at index date (a confirmed diagnosis and ≥1 DPC encounter during the 6 months before the index date). DPC, diagnostic procedure combination; GPP, generalized pustular psoriasis

This was a retrospective cohort study using data obtained from the Japanese MDV database, a national acute hospital‐based claims database that includes inpatients and outpatients under the diagnostic procedure combination (DPC) system, including diagnoses identified according to the International Classification of Diseases, 10^th^ revision (ICD‐10) (as dictated by the World Health Organization), and standard disease codes (named by the Japanese Ministry of Health, Labour, and Welfare). Medications were identified through prescriptions containing generic or brand names of medication submitted on health insurance claims. The study period was between July 1, 2014 and December 31, 2019. The patient selection period was January 1, 2015 to December 31, 2019: patients could join the study at any time after January 1, 2015, once they had fulfilled the study eligibility criteria (index date) (Figure 1). The index date was the date of the first inpatient or outpatient claim with a diagnosis code of L40.0 or L40.1, respectively, with a preceding eligible 6‐month baseline period. A data collection follow‐up period of 12 months started on the index date.

**FIGURE 2:**
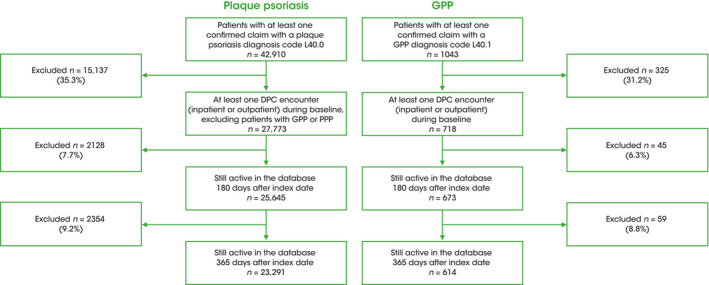
Study population. DPC, diagnostic procedure combination; GPP, generalized pustular psoriasis; PPP, palmoplantar pustulosis

Three cohorts were evaluated: patients with GPP (with an ICD‐10 code of L40.1), patients with plaque psoriasis (with an ICD‐10 code of L40.0), and a general population comparator control cohort, matched to patients with GPP (4:1) based on age (patients aged <95 years were matched exactly on age; patients aged ≥95 years were age group matched) and sex at the index month, without a diagnosis of GPP (L40.1), palmoplantar pustulosis (L40.3), or psoriasis (but allowing for a diagnosis of psoriatic arthritis) according to ICD‐10 codes L40, L40.0, L40.2, L40.4, L40.8, and L40.9. Patients with GPP and concomitant plaque psoriasis were included in the GPP cohort. The comparator general population cohort is denoted throughout as the matched control cohort. Only patients with a confirmed diagnosis and at least one interaction with the DPC system in the 6 months before the index date were eligible for inclusion. All patients in the GPP and plaque psoriasis cohorts must still be active in the database after 12 months (365 days) post index date to be included in the 12‐month follow‐up analyses (Figure 2). For all ICD‐10 codes and medications utilized, see Tables S1─S4. Lower and upper age limits were not applied.

All analyses were performed using the Instant Health Data (IHD) software (Panalgo, Boston, MA, USA) and R software, version 3.5.2 (R Foundation for Statistical Computing, Vienna, Austria). No comparative statistical analysis was undertaken; descriptive statistics are used throughout, including the mean and standard deviation (SD), median and interquartile range (IQR) for continuous variables, and frequencies and percentages for categorical variables. For HCRU analyses, all‐cause visits were included.

This study was approved by the ethics committee of Yoyogi Mental Clinic (approval number: NBI207‐2). The study was conducted according to the Declaration of Helsinki.

## 3 RESULTS

### 3.1 Patient demographics and characteristics at baseline

A total of 718 patients with GPP and 27,773 patients with plaque psoriasis who had at least one DPC recorded event during the baseline period were identified. A matched control cohort of 2867 patients was also identified (Figure 2). Patient characteristics and demographics at baseline are shown in Table 1. There were slightly more female (51.5%) than male patients with GPP. In contrast, in the plaque psoriasis cohort, a minority of patients were female (38.7%). The mean ages of patients with GPP and plaque psoriasis were 60.9 and 62.9 years, respectively (Table 1). A higher proportion of patients with GPP (53.1%) visited a large hospital (≥500 beds) than those with plaque psoriasis (40.8%) (Table 1).

**TABLE 1:** Baseline demographics and characteristics

	GPP *n* = 718	Plaque psoriasis *n* = 27,773	Matched control cohort *n* = 2867
Male, *n* (%)	348 (48.5)	17,017 (61.3)	1387 (48.4)
Female, *n* (%)	370 (51.5)	10,756 (38.7)	1480 (51.6)
Age, years, mean (SD)	60.9 (18.2)	62.9 (16.9)	60.9 (18.2)
<18 years, *n* (%)	17 (2.4)	484 (1.7)	68 (2.4)
18‐64 years, *n* (%)	342 (47.6)	12,167 (43.8)	1368 (47.7)
≥65 years, *n* (%)	359 (50.0)	15,122 (54.5)	1431 (49.9)
BMI in patients with at least one record, kg/m^2^, mean (SD)	23.5 (5.7)^a^	23.9 (6.4)^a^	22.9 (5.2)^a^
Range of number of beds of hospital patient visits, *n* (%)			
≤199 beds	29 (4.0)	2145 (7.7)	310 (10.8)
200–499 beds	308 (42.9)	14,288 (51.5)	1661 (57.9)
≥500 beds	381 (53.1)	11,340 (40.8)	896 (31.3)

Abbreviations: BMI, body mass index; GPP, generalized pustular psoriasis; SD, standard deviation.

^a^ Missing patients are not included in these analyses.

### 3.2 Comorbidities during the 12‐month follow‐up period

**FIGURE 3:**
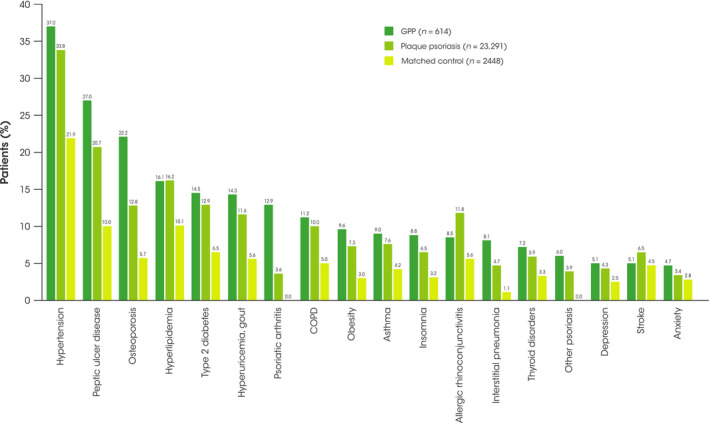
Most common comorbidities in patients with GPP during the 12‐month follow‐up period. COPD, chronic obstructive pulmonary disease; GPP, generalized pustular psoriasis

During the 12‐month follow‐up period, patients with GPP had a higher proportion of diagnoses for comorbidities compared with patients with plaque psoriasis or the matched control cohort. The most‐diagnosed comorbidities in patients with GPP included psoriatic arthritis (12.9% vs. 3.6% and 0.0%, respectively), other forms of psoriasis (6.0% vs. 3.9% and 0.0%), peptic ulcer disease (27.0% vs. 20.7% and 10.0%), osteoporosis (22.1% vs. 12.8% and 5.7%), and interstitial pneumonia (8.1% vs. 4.7% and 1.1%). Other comorbidities more commonly diagnosed in patients with GPP than those with plaque psoriasis and the matched control cohort included, hypertension, type 2 diabetes, hyperuricemia, chronic obstructive pulmonary disease, asthma, obesity, and insomnia (Figure 3).

### 3.3 Comorbidities by age

Many of these comorbidities were generally more common in patients who were aged ≥65 years than in those who were aged 18–64 years, irrespective of the disease cohort. However, whereas the frequency of type 2 diabetes was similar in patients with GPP or plaque psoriasis who were aged 18–64 years, the frequency substantially increased in elderly patients with GPP compared with those with plaque psoriasis (GPP vs plaque psoriasis and matched control cohort, respectively: 18–64 years, 8.4% vs. 10.2% and 4.9%, vs. ≥65 years, 21.0% vs. 15.4% and 8.5%). Comorbidities that were less frequent in elderly patients included psoriatic arthritis and interstitial pneumonia.

### 3.4 Dermatologic medication use during the 12‐month follow‐up period

Overall, during the 12‐month follow‐up period, patients with GPP were slightly more likely than patients with plaque psoriasis to receive any treatment of topical steroids, non‐biologic systemic, or biologic therapies (88.4% vs. 79.7%). Fewer patients with GPP received topical steroid therapy alone than those with plaque psoriasis (14.0% vs. 42.0%), whereas more patients with GPP received topical steroids and non‐biologic systemic combination therapy (42.3% vs. 23.9%) than those with plaque psoriasis. In addition, patients with GPP were as likely to receive biologic monotherapy (0.8% vs. 0.9%) and non‐biologic monotherapy (9.8% vs 9.2%) as those with plaque psoriasis (Table 2). However, patients with GPP were more likely to receive biologic therapies in combination with topical steroids or non‐biologic therapies than patients with plaque psoriasis (21.5% vs. 3.8%) (Table 2).

**TABLE 2:** Systemic drug use during the 12‐month follow‐up period on or after the qualifying claim date

Drug, *n* (%)	GPP *n* = 614	Plaque psoriasis *n* = 23,291
No treatment (topical steroids, non‐biologic systemic, and biologic)	71 (11.6)	4719 (20.3)
Topical steroid monotherapy	86 (14.0)	9773 (42.0)
Non‐biologic systemic monotherapy	60 (9.8)	2147 (9.2)
Biologic monotherapy	5 (0.8)	210 (0.9)
Topical steroids + non‐biologic systemic	260 (42.3)	5557 (23.9)
Topical steroids + biologic	29 (4.7)	301 (1.3)
Non‐biologic systemic + biologic	22 (3.6)	171 (0.7)
Topical steroids + non‐biologic systemic + biologic	81 (13.2)	413 (1.8)

Note

All medication groups are mutually exclusive.

Abbreviation: GPP, generalized pustular psoriasis.

**FIGURE 4:**
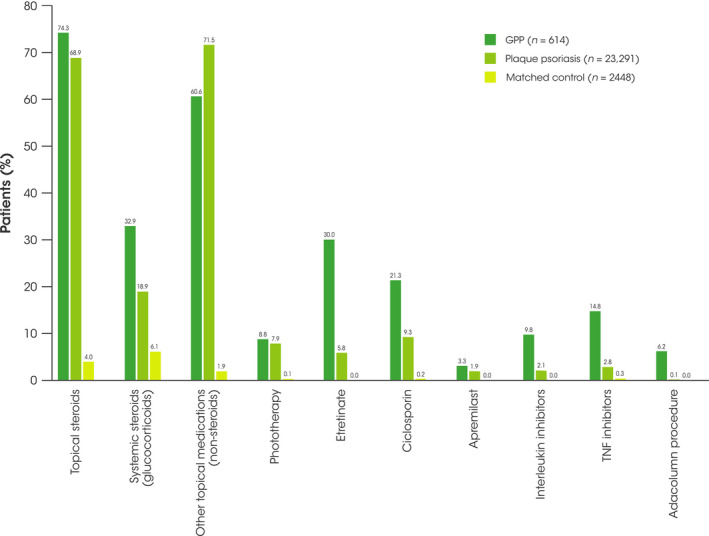
Dermatologic medication use during the 12‐month follow‐up period on or after the qualifying claim date. GPP, generalized pustular psoriasis; TNF, tumor necrosis factor

Patients with GPP were more likely to receive topical steroids (multiple), systemic steroids, tumor necrosis factor inhibitors, and interleukin inhibitors than those with plaque psoriasis (Figure 4). Of the biologic therapies, the most commonly prescribed medication in patients with GPP was infliximab (9.9%); the most commonly prescribed biologic in patients with plaque psoriasis was ustekinumab (1.5%). The most commonly prescribed non‐biologic therapies in patients with GPP were etretinate (30.0%), ciclosporin (21.3%), and methotrexate (7.0%). Patients with plaque psoriasis were less often treated with etretinate (5.8%), ciclosporin (9.3%), and methotrexate (2.6%), although these were still the most frequently prescribed medications for this cohort. In addition, apremilast was prescribed in a higher proportion of patients with GPP (3.3%) than in patients with plaque psoriasis (1.9%). Of the topical medications, maxacalcitol was the most frequently prescribed medication in patients with GPP (26.6%); in addition, maxacalcitol was more likely to be prescribed in those with plaque psoriasis (32.5%). More patients with GPP received phototherapy than those with plaque psoriasis (8.8% vs. 7.9%), with 6.2% of patients with GPP also received apheresis with Adacolumn, compared with 0.1% of those with plaque psoriasis.

### 3.5 Medication use by age in patients with GPP

When stratified, patients with GPP of all age groups were as likely to receive topical steroids and topical non‐steroids; however, patients aged ≥65 years were slightly more likely than those who were aged 18–64 years to receive systemic steroids (35.7% vs. 31.3%). In contrast, patients who were aged 18–64 years were more likely to receive biologic systemic therapies than those who were aged ≥65 years (35.0% vs. 10.7%). Of the biologic therapies, patients who were aged 18–64 years were more likely to receive tumor necrosis factor inhibitors (24.9% vs. 5.7%), specifically adalimumab (6.7% vs. 3.0%) and infliximab (18.5% vs. 2.0%), and interleukin inhibitors (14.1% vs. 5.7%), specifically ustekinumab (5.4% vs. 1.7%) and secukinumab (7.4% vs. 2.0%). Patients aged ≥65 years were more likely to received etretinate than those aged 18–64 years (36.7% vs. 24.6%).

### 3.6 Concomitant medication use during the 12‐month follow‐up period

**FIGURE 5:**
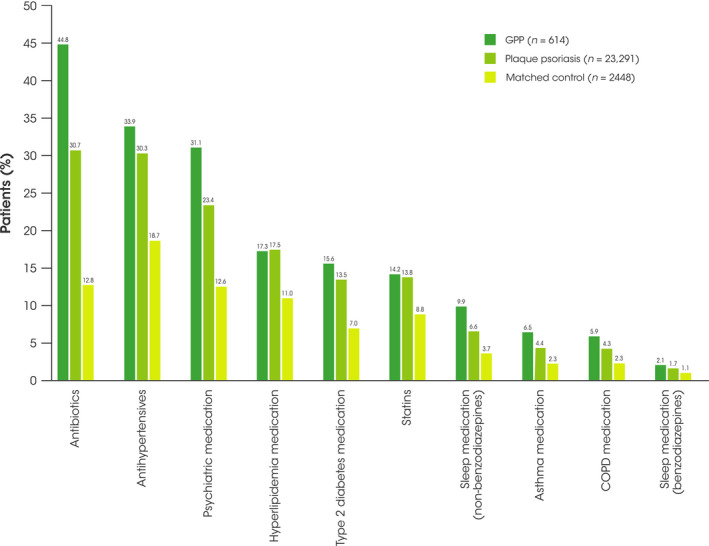
Most common concomitant medication burden during the 12‐month follow‐up period. COPD, chronic obstructive pulmonary disease; GPP, generalized pustular psoriasis

During the 12‐month follow‐up period, a higher proportion of patients with GPP received concomitant medication for comorbidities than patients with plaque psoriasis and the matched control cohort. These included antihypertensive medication, psychiatric medication, antibiotics, and non‐benzodiazepine medication (Figure 5).

### 3.7 All‐cause HCRU during the 12‐month follow‐up period

The mean number of outpatient visits for patients with GPP was higher than in the plaque psoriasis and matched control cohorts (mean [SD]: 15.7 [15.0] vs. 13.8 [16.1] and 7.5 [10.6], respectively) (Table 3). Patients with GPP were also more likely to require inpatient hospitalization that was >24 hours in duration compared with both the plaque psoriasis and matched control cohorts (35.5% vs. 23.0% and 11.6%) (Table 3), whereas a very low percentage in any cohort had inpatient hospitalizations that were ≤24 hours in duration. The overall mean duration of hospitalization, in days, was longer in the GPP cohort than in the plaque psoriasis or the matched control cohorts (mean [SD]: 25.2 [27.7] vs. 22.0 [37.2] and 19.3 [28.2]) (Table 3). Age stratification showed that hospitalization was higher in patients with GPP across all age groups.

**TABLE 3:** All‐cause HCRU and medical costs during the 12‐month follow‐up period

	GPP *n* = 614	Plaque psoriasis *n* = 23,291	Matched cohort *n* = 2448
All‐cause outpatient visits, *n* (%)	591 (96.3)	22,981 (98.7)	1727 (70.5)
Median (IQR)	13 (7–19)	10 (6–17)	5 (2–9)
Mean (SD)	15.7 (15.0)	13.8 (16.1)	7.5 (10.6)
All‐cause inpatient visits,^a^ *n* (%)	223 (36.3)	5436 (23.3)	289 (11.8)
All‐cause inpatient visits^a^ ≤24 h, *n* (%)	5 (0.8)	115 (0.5)	10 (0.4)
All‐cause inpatient visits^a^ >24 h, *n* (%)	218 (35.5)	5366 (23.0)	283 (11.6)
Median visits per patient (IQR)	1 (1–2)	1 (1–2)	1 (1–2)
Mean visits per patient (SD)	1.8 (1.3)	1.7 (1.4)	1.5 (1.6)
Duration of inpatient visits^a^ >24 h, days			
Median (IQR)	17 (8–35)	12 (6–24)	11 (5–22)
Mean (SD)	25.2 (27.7)	22.0 (37.2)	19.3 (28.2)
Outpatient costs, ¥ ×10^3^, mean (SD)			
Prescription costs	781.5 (1180.6)	367.1 (981.0)	160.0 (648.8)
Medical costs	213.3 (464.1)	145.0 (388.4)	80.4 (258.2)
Inpatient costs, ¥ ×10^3^, mean (SD)			
Prescription costs	461.5 (1190.5)	256.0 (906.1)	121.9 (261.8)
Medical costs	1594.7 (1770.7)	1339.0 (1539.0)	1111.6 (1190.3)
Total costs, ¥ ×10^3^, mean (SD)			
Prescription	901.1 (1360.6)	415.0 (1088.2)	104.9 (534.7)
Medical	776.7 (1384.3)	453.8 (1050.8)	187.1 (598.6)
Overall total costs, ¥ ×10^3^, mean (SD)^b^	1677.8 (2145.2)	868.7 (1679.0)	292.0 (887.6)

Abbreviations: GPP, generalized pustular psoriasis; HCRU, healthcare resource utilization; IQR, interquartile range; SD, standard deviation.

^a^ Inpatient visits are reported for patients with at least one inpatient claim during the 12‐month follow‐up period.

^b^ Overall total costs = total prescription costs + total medical costs.

Overall, patients with GPP had the highest all‐cause hospitalization costs (mean [SD]: GPP, ¥1.67 million [2.14 million]; plaque psoriasis, ¥868.7K [1.68 million]; matched cohort, ¥292.0K [887.6K]) (Table 3). Stratification for prescription and medical costs showed that patients with GPP had higher prescription and medical costs than both the plaque psoriasis and matched cohorts, in both outpatient and inpatient settings (Table 3).

The most common reason for outpatient and inpatient visits in both patients with GPP and patients with plaque psoriasis was psoriasis (under claim code L40); however, a higher proportion of patients with GPP required inpatient hospital visits than those with plaque psoriasis (81.6% vs. 46.7%, respectively; Table 4), whereas a similar proportion of patients with GPP and with plaque psoriasis required outpatient visits (95.5% and 97.4%, respectively; Table 5). Other common reasons for inpatient hospitalizations included essential primary hypertension and gastro‐esophageal reflux disease (Table 4); reasons for outpatient visits included dermatitis (other) and epidermal thickening (other) (Table 5). The proportions for the most common diagnostic reasons for inpatient hospitalization were similar in patients with GPP or plaque psoriasis. A lower proportion of the matched cohort had inpatient and outpatient hospital claims for the most common ailments compared with patients with GPP and patients with plaque psoriasis, with the exception of claims for disorders of lipoprotein and other lipidemias, for which the proportion was similar to those with GPP (Tables 4 and 5).

**TABLE 4:** Top 15 reasons for inpatient hospitalizations (based on the first three digits of the ICD‐10 code^a^) experienced by patients with GPP during the 12‐month follow‐up period

ICD‐10 diagnostic code^a^	Diagnosis, *n* (%)	GPP *n* = 614	Matched control cohort *n* = 2448	ICD‐10 diagnostic code^a^	Diagnosis, *n* (%)	Plaque psoriasis *n* = 23,291
	Total number of patients with at least one inpatient diagnostic claim	*n* = 228	*n* = 314		Total number of patients with at least one inpatient diagnosis claim	*n* = 5561
L40	Psoriasis	186 (81.6)	0 (0.0)	L40	Psoriasis	2599 (46.7)
I10	Essential (primary) hypertension	82 (36.0)	113 (36.0)	I10	Essential (primary) hypertension	2349 (42.2)
K21	Gastro‐esophageal reflux disease	70 (30.7)	62 (19.7)	E11	Non‐insulin‐dependent diabetes mellitus	1569 (28.2)
E11	Non‐insulin‐dependent diabetes mellitus	63 (27.6)	73 (23.2)	K21	Gastro‐esophageal reflux disease	1551 (27.9)
K59	Other functional intestinal disorders^b^	59 (25.9)	72 (22.9)	K59	Other functional intestinal disorders^b^	1492 (26.8)
K29	Gastritis and duodenitis	49 (21.5)	41 (13.1)	E78	Disorders of lipoprotein metabolism and other lipidemias	1213 (21.8)
M54	Dorsalgia	48 (21.1)	31 (9.9)	G47	Sleep disorders	1046 (18.8)
G47	Sleep disorders	45 (19.7)	55 (17.5)	I50	Heart failure	943 (17.0)
K25	Gastric ulcer	45 (19.7)	38 (12.1)	K25	Gastric ulcer	921 (16.6)
E78	Disorders of lipoprotein and other lipidemias	43 (18.9)	59 (18.8)	K29	Gastritis and duodenitis	835 (15.0)
L30	Other dermatitis	43 (18.9)	9 (2.9)	D50	Iron deficiency anemia	603 (10.8)
M81	Osteoporosis without pathological fracture	40 (17.5)	26 (8.3)	N/A	N/A	N/A
L85	Other epidermal thickening	39 (17.1)	9 (2.9)	N/A	N/A	N/A
I50	Heart failure	38 (16.7)	47 (15.0)	N/A	N/A	N/A
D50	Iron deficiency anemia	33 (14.5)	36 (11.5)	N/A	N/A	N/A

Abbreviations: GPP, generalized pustular psoriasis; ICD‐10, International Classification of Diseases, 10th revision; MDV, Medical Data Vision; N/A, not applicable; WHO, World Health Organization.

^a^ ICD‐10 codes within the MDV database follow WHO classification.

^b^ ICD‐10 code K59 can include, but does not differentiate, changes in bowel habit, intestinal malabsorption, psychogenic intestinal disorders, functional disorders of the stomach, and constipation.

**TABLE 5:** Top 15 reasons for outpatient visits (based on the first three digits of the ICD‐10 code^a^) experienced by patients with GPP during the 12‐month follow‐up period

ICD‐10 diagnostic code^a^	Diagnosis, *n* (%)	GPP *n* = 614	Matched control cohort *n *= 2448	ICD‐10 diagnostic code^a^	Diagnosis, *n* (%)	Plaque psoriasis *n *= 23,291
	Total number of patients with at least one outpatient diagnostic claim	*n* = 601	*n* = 1758		Total number of patients with at least one outpatient diagnostic claim	*n* = 23,077
L40	Psoriasis	574 (95.5)	4 (0.2)	L40	Psoriasis	22,482 (97.4)
L30	Other dermatitis	284 (47.3)	136 (7.7)	L30	Other dermatitis	10,119 (43.8)
L85	Other epidermal thickening	284 (47.3)	107 (6.1)	L85	Other epidermal thickening	9619 (41.7)
I10	Essential (primary) hypertension	209 (34.8)	511 (29.1)	I10	Essential (primary) hypertension	7405 (32.1)
K21	Gastro‐esophageal reflux disease	177 (29.5)	348 (19.8)	K21	Gastro‐esophageal reflux disease	6384 (27.7)
M54	Dorsalgia	177 (29.5)	278 (15.8)	E78	Disorders of lipoprotein and lipidemias	5800 (25.1)
K29	Gastritis and duodenitis	171 (28.5)	353 (20.1)	K29	Gastritis and duodenitis	5617 (24.3)
E78	Disorders of lipoprotein and other lipidemias	157 (26.1)	390 (22.2)	K59	Other functional intestinal disorders^b^	5292 (22.9)
K25	Gastric ulcer	149 (24.8)	220 (12.5)	B35	Dermatophytosis	5156 (22.3)
K59	Other functional intestinal disorders^b^	142 (23.6)	336 (19.1)	M54	Dorsalgia	4816 (20.9)
E14	Unspecified diabetes mellitus	140 (23.3)	247 (14.1)	K25	Gastric ulcer	4379 (19.0)
B35	Dermatophytosis	135 (22.5)	47 (2.7)	G47	Sleep disorders	4347 (18.8)
G47	Sleep disorders	132 (22.0)	258 (14.7)	E14	Unspecified diabetes mellitus	4323 (18.7)
M81	Osteoporosis without pathological fracture	124 (20.6)	136 (7.7)	N/A	N/A	N/A
L08	Other local infections of skin and subcutaneous tissue	106 (17.6)	21 (1.2)	N/A	N/A	N/A

Abbreviations: GPP, generalized pustular psoriasis; ICD‐10, International Classification of Diseases, 10th revision; MDV, Medical Data Vision; N/A, not applicable; WHO, World Health Organization.

^a^ ICD‐10 codes within the MDV database follow WHO classification.

^b^ ICD‐10 code K59 can include, but does not differentiate, changes in bowel habit, intestinal malabsorption, psychogenic intestinal disorders, functional disorders of the stomach, and constipation.

## 4 DISCUSSION

This study describes the clinical characteristics of patients with GPP in Japan included in the MDV database. The MDV database covers health claims data from over 400 hospitals managing acute care in Japan (accounting for ~23% of hospitals managing acute care) and contains data for over 30 million patients, 34% of whom are aged ≥65 years. Patients with GPP with a moderate‐to‐severe profile (Japanese Dermatological Association severity index score ≥7) who qualified for an intractable disease designation, as well as qualifying patients who received continuous treatment (e.g. biologics) and displayed mild disease, were covered by national insurance for their medical expenses associated with GPP.

Patients with GPP or plaque psoriasis experienced more comorbidities than patients in the matched control cohort; these comorbidities were generally consistent with those frequently observed in GPP studies conducted in Asia, Africa, and Europe.^3^, ^4^, ^12^, ^13^ In the GPP and plaque psoriasis cohorts, the prevalence of hypertension, type 2 diabetes, and hyperlipidemia were higher compared with the matched control cohort. In this article, we show that patients with GPP were more likely to experience most comorbidities measured in this study versus patients with plaque psoriasis, including comorbidities often associated with plaque psoriasis (e.g. psoriatic arthritis, osteoporosis),^17^ as well as those associated with biologic use in patients with psoriasis (e.g. interstitial pneumonia).^18^ This suggests that patients with GPP experience a higher burden of illness than those with plaque psoriasis. The higher prevalence of specific comorbidities in those aged ≥65 years versus the younger cohort is not surprising. The risk of becoming overweight and developing type 2 diabetes and hypertension increases with increasing age. Interestingly, although comorbidities associated with obesity are more prevalent than those not associated with obesity, obesity itself is not reported as a common comorbidity in this study, despite it being a predisposing risk factor for multiple psoriatic diseases.^19^ This could be because obesity is not sufficiently recorded by the MDV database and data are not available for all the participants included in this study, demonstrating a limitation of the MDV database.

Types of medications prescribed for comorbidities were generally consistent with the prevalence of comorbidities in patients with GPP; however, it is not possible to confirm which medications were prescribed for specific comorbidities. Because diagnosis codes were not linked to prescriptions for medications, determining the reasons for prescribing patterns is difficult. The Japanese insurance system prohibits physicians from prescribing medications without a confirmed diagnosis, suggesting that those medications were likely prescribed for the treatment of other conditions that are not captured by the database. For example, increased prescription of psychiatric medication may be because of increased psychiatric disorders outside of the parameters of this study, given that 31.1% of patients with GPP received a prescription for psychiatric medication, although only 12.1% of patients with GPP overall had a diagnostic code for attention deficit disorder, anxiety, or depression. Antibiotics were more likely to be prescribed in the GPP than plaque psoriasis cohort. Although infection was among the 15 most common reasons for outpatient visits in patients with GPP, the specific reasons for antibiotic prescriptions are unknown. However, GPP is sometimes triggered by infection,^20^ and in Japan, it is common practice to prescribe antibiotics for the purpose of preventing a GPP flare that may be triggered by an infection, or for treating infections as a result of pustules on the skin. The slightly increased use of medications for asthma and chronic obstructive pulmonary disease in patients with GPP versus those with plaque psoriasis may also be indicative of a higher prevalence of lung and respiratory infections. This is consistent with a higher prevalence of interstitial pneumonia in the GPP patient population compared with the plaque psoriasis population. Another highly prescribed class of medication was antihypertensives. The most common diagnosis claims for inpatient visits confirm that hypertension and disorders of metabolism are among the most common reasons for inpatient hospitalization, which may explain why antihypertensives are frequently prescribed in patients across all cohorts studied.

Patients with GPP were more often treated with biologic and non‐biologic systemic therapies than those with plaque psoriasis. Although biologics and non‐biologic systemic therapies are expensive, if a patient qualified for intractable disease designation, the cost of these medications was covered by national insurance increasing their accessibility; however, those who do not qualify, are required to cover the expenses themselves.

It would be interesting to investigate the regimens of treatments received, since this may indicate disease severity.^21^ Although it is not possible to evaluate disease severity from this database, demonstrating a limitation of this study, disease severity may be deduced from the proportion of patients receiving systemic treatments. In this study, almost twice as many patients with GPP (74.4%) than those with plaque psoriasis (37.8%) received systemic treatments, suggesting that those with GPP were more likely to experience severe disease than those with plaque psoriasis. Those with plaque psoriasis may have been more likely to display a heterogeneous population of patients experiencing mild, moderate, and severe disease. It is also possible that topical medications alone, which are often prescribed for plaque psoriasis, may not be adequate to manage the skin and systemic symptoms of those with acute flares of GPP. Nearly one‐third of patients with GPP received systemic steroid treatment, alone or in combination with other therapies, which was especially high compared with its use in patients with plaque psoriasis and the matched control cohort. This was of particular interest given that tapering or withdrawal of systemic corticosteroids can induce pustules, and that current treatment guidelines do not recommend systemic steroids alone for the treatment of GPP in Japan, highlighting an unmet need in GPP treatment.^2^ The high use of systemic steroids may also explain why there is a higher prevalence of osteoporosis among patients with GPP compared with those with plaque psoriasis, because systemic steroid use is associated with an adverse effect on bone mass and the onset of osteoporosis. Older patients (aged ≥65 years) were less likely to receive biologic treatments than those aged 18–64 years. This could be due to a reluctance to treat older patients with biologic drugs that suppress the immune system, because older patients are more likely to be immunocompromised than younger patients. Of note, although prescription of anti‐tumor necrosis factor or anti‐interleukin‐17A/receptor A biologics is difficult due to the greater risk of infection in older patients with GPP, the use of anti‐interleukin‐23 biologics could be higher than the numbers reflected in this study because these were approved in 2018.

Overall, the MDV database showed that patients with GPP and those with plaque psoriasis were often hospitalized for similar reasons; however, more patients with GPP required inpatient care and the overall duration of hospitalization was longer than for those with plaque psoriasis or the matched cohort. This resulted in higher healthcare costs than for those with plaque psoriasis. Reasons for inpatient hospitalizations concurred with the overall proportions of comorbidities experienced, specifically hypertension, gastrointestinal disorders, and metabolic disorders. The prevalence of some gastrointestinal disorders may be due to stress,^22^ with gastrointestinal bleeding and perforation also being associated with the use of oral steroids in hospitalized patients,^23^ although it is not clear what the cause of gastrointestinal disorders was in our study. The diagnosis claim code for inpatient and outpatient hospitalization for psoriasis was limited to the classification of psoriasis (L40), so it is unknown whether patients were hospitalized specifically because of their condition, i.e. GPP (L40.1) or plaque psoriasis (L40.0). Given that nearly twice as many patients with GPP required inpatient hospitalization under the diagnosis code L40, compared with those with plaque psoriasis, and no patients in the matched cohort were hospitalized under this claim code, it seems likely that patients with GPP were more likely to be hospitalized because of their disease compared with those with plaque psoriasis.

There are several limitations to this study. Only patients with a claim within the DPC hospital system were captured. Patient tracking is terminated if they do not return to the same DPC hospital, or are transferred to another hospital or clinic outside of the DPC system. Therefore, these patients would have been excluded from the study dataset, with no further data captured. Owing to the large imbalances introduced by the disparities in sample sizes between the GPP and plaque psoriasis cohorts, it was not possible to statistically conclude that patients with GPP experience more severe disease with poorer outcomes than those with plaque psoriasis. The lack of a validation algorithm for positive case identification is also a limitation of this study; however, the standard criteria for identifying patients with GPP were used. The MDV database only contains information provided by hospitals applying the flat‐fee payment system, which are mostly large hospitals responsible for acute care. Therefore, a substantial proportion of the patients included were likely in poor health compared with the average population. In addition, these data do not include patients who received care outside of the DPC hospital, therefore data are limited to those receiving care within a DPC hospital only. This study does not include vital signs or laboratory measurements, which could add context to the systemic symptoms that can be present in patients with GPP or plaque psoriasis. In the MDV database, approximately 45% of the population is aged ≥65 years, compared with 26.6% of the overall population in Japan (as the population was in 2015),^24^ which may affect the generalizability of the study. Also, the average age of those with GPP within the MDV database is higher than the epidemiologic data of patients with GPP in Asia suggests (i.e. 60.9 years vs. 40.0–50.0 years, respectively).^13^ This, as well as the complexity of treating an older population, could impact the perceived severity of the disease, including the prevalence of comorbidities and potential contraindications between treatments.

Overall, the MDV database has enabled the assessment of long‐term disease management and clinical characterization of patients with GPP. We have shown that, in Japan, patients with GPP have a higher burden of illness, including a higher prevalence of comorbidities and medication burden, than those with plaque psoriasis, as well as greater HCRU. It is hoped that these results, in addition to further investigation, will reinforce the need for a distinct treatment and management pathway for patients with GPP and will encourage the further development of disease management practices for patients with GPP.

## CONFLICTS OF INTEREST

R Gao, N Kotowsky, and R Shimizu are employees of Boehringer Ingelheim. A Morita declares receiving research grants, consulting fees, and/or speaker's fees from AbbVie, Boehringer Ingelheim, Celgene, Eli Lilly, Eisai, Janssen, Kyowa Kirin, LEO Pharma, Maruho, Mitsubishi Tanabe, Nichi‐Iko, Nippon Kayaku, Novartis, Sun Pharmaceutical Industries, Taiho Pharmaceutical, Torii Pharmaceutical, and Ushio. Y Okubo declares receiving research grants from Eisai, Torii, Maruho, and Shiseido, and has current consulting/advisory board agreements and/or speakers bureau and/or clinical trials from AbbVie, Amgen, Boehringer Ingelheim, Bristol‐Myers Squibb, Celgene, Eisai, Eli Lilly, Janssen Pharma, JIMRO, Kyowa Kirin, LEO Pharma, Maruho, Novartis, Pfizer, Sanofi, Sun Pharma, Taiho, Tanabe‐Mitsubishi, Torii, and UCB Pharma.

## Supporting information

Table S1‐S4
