# Supplementary material for: Patient characteristics and burden of disease in Japanese patients with generalized pustular psoriasis: Results from the Medical Data Vision claims database
Source: J Dermatol. 2021 Jul 1;48(10):1463–73. doi: 10.1111/1346-8138.16022 (PMC9291902; doi:10.1111/1346-8138.16022)
Supplement: Supplementary file 1 — Table S1‐S4 [file JDE-48-1463-s001.docx]

**Supplementary Materials**

**Title:** Patient characteristics and burden of disease in Japanese patients with generalized pustular psoriasis: results from the Medical Data Vision claims database

**Corresponding author:** Nirali Kotowsky

**Content**

**Tables:**

Supplementary Table S1. List of comorbidities

Supplementary Table S2. List of dermatologic medications

Supplementary Table S3. List of medications for comorbidities

Supplementary Table S4. Added medications of interest

**Supplementary Table S1. List of comorbidities**

| **Comorbidity** | **ICD-10 codes**† |
| --- | --- |
| **Allergies** |  |
| Allergic rhinoconjunctivitis | J30.4 |
| Allergic contact dermatitis | L23 |
| **Infections** |  |
| Tonsillitis | Standard disease name |
| Sinusitis | Standard disease name |
| **Autoimmune conditions** |  |
| Psoriatic arthritis | L40.5 |
| Other psoriasis | L40.2, L40.4, L40.8, L40.9 |
| Pustulotic artho-osteitis | Standard disease code: 8845161 |
| **Dental conditions** |  |
| Apical periodontal | Standard disease name |
| Periodontitis | Standard disease name |
| Alveolar pyorrhea | Standard disease name |
| **Bone conditions** |  |
| Osteoporosis | M80.x, M81.x, M82.x |
| **Cardiovascular conditions** |  |
| Myocardial infarction | I21.0, I21.1, I21.2, I21.3, I21.4, I21.9, I21.A, I22.x |
| Stroke | I60.0, I60.1, I60.2, I60.3, I60.4, I60.5, I60.6, I60.7, I60.8, I60.9, I61.0, I61.1, I61.2, I61.3, I61.4, I61.5, I61.6, I61.8, I61.9, I62.0, I62.1, I62.9, I63.0, I63.1, I63.2, I63.3, I63.4, I63.5, I63.6, I63.8, I63.9 |
| Hypertension | I10.x – I15.x |
| **Gastrointestinal conditions** |  |
| Celiac disease | K90.0 |
| Crohn’s disease | K50.0, K50.1, K50.8, K50.9 |
| Diverticulitis | K57.0, K57.1, K57.2, K57.3, K57.4, K57.5, K57.8, K57.9 |
| Peptic ulcer disease | K25.0, K25.1, K25.2, K25.3, K25.4, K25.5, K25.6, K25.7, K25.9, K26.0, K26.1, K26.2, K26.3, K26.4, K26.5, K26.6, K26.7, K26.9, K27.0, K27.1, K27.2, K27.3, K27.4, K27.5, K27.6, K27.7, K27.9, K28.0, K28.1, K28.2, K28.3, K28.4, K28.5, K28.6, K28.7, K28.9 |
| Ulcerative colitis | K51.0, K51.2, K51.3, K51.4, K51.5, K51.8, K51.9 |
| **Eye conditions** |  |
| Uveitis | Standard disease name  3643001 – uveitis  3601002 – sympathetic uveitis  3601004 – panuveitis  3631001 – peripheral uveitis  3640001 – viral uveitis  3640009 – secondary uveitis  3643025 – acute anterior uveitis  3643026 – Kirisawa type uveitis  3643027 – intermediate uveitis  8830189 – subacute anterior uveitis  8830399 – allergic uveitis  8831419 – suppurative uveitis  8833078 – tuberculous uveitis  8834554 – peripheral uveitis  8838292 – endogenous uveitis  8838368 – intractable uveitis  8839042 – recurrent anterior uveitis  8839834 – keratouveitis  8839984 – herpesviral uveitis  8844005 – HTLV-1 uveitis |
| **Hepatic conditions** |  |
| Non-alcoholic fatty liver disease | K76.0 |
| Hepatic failure | K72, K72.0, K72.1, K72.9 |
| **Hormonal/metabolic conditions** |  |
| Type 2 diabetes | E11.0, E11.1, E11.2, E11.3, E11.4, E11.5, E11.6, E11.8, E11.9, E14.0, E14.1, E14.2, E14.3, E14.4, E14.5, E14.6, E14.7, E14.8, E14.9  AND at least one anti-diabetic treatment (insulin included) to occur within 30 days before or after the diagnosis code |
| Hyperlipidemia | E78.0, E78.1, E78.2, E78.3, E78.4, E78.5  AND at least one hyperlipidemia treatment |
| Metabolic syndrome | E88.81 |
| Obesity | E66.0, E66.3, E66.8, E66.9  OR BMI≥25 calculated from height and weight |
| Hyperuricemia | Standard disease name |
| Thyroid disorders | Standard disease name |
| **Pulmonary conditions** |  |
| Asthma | J45 |
| Chronic obstructive pulmonary disease | J40, J41.0, J41.1, J41.8, J42, J43.0, J43.1, J43.2, J43.8, J43.9, J44.0, J44.1, J44.9, J47.0, J47.1, J47.9 |
| Interstitial pneumonia | Standard disease name |
| **Psychiatric conditions** |  |
| ADHD | F90 |
| Anxiety | F06.4, F40.0, F40.1, F40.2, F40.8, F40.9, F41.0, F41.1, F41.3, F41.8, F41.9, F43.22, F43.23, F93.0 |
| Depression | F32, F33 |
| Schizophrenia | Standard disease name |
| Suicide ideation | SDC codes: 3009015, 3009016 |
| **Sleep disorders** |  |
| Sleep apnea | G47.3 |
| Insomnia | G47.0  AND at least one insomnia treatment |
| **Renal conditions** |  |
| Acute renal failure | N17.0, N17.1, N17.2, N17.8, N17.9, N19 |
| Chronic kidney disease | N18.2, N18.3, N18.4, N18.5, N18.9, N18.1, I12, I12.0, I12.9, I13, I13.0, I13.1, I13.2, I13.9, N03, N03.0, N03.1, N03.2, N03.3, N03.4, N03.5, N03.6, N03.7, N03.8, N03.9, N04, N04.0, N04.1, N04.2, N04.3, N04.4, N04.5, N04.6, N04.7, N04.8, N04.9, N05, N05.0, N05.1, N05.2, N05.3, N05.4, N05.5, N05.6, N05.7, N05.8, N05.9, N08, N08.0, N08.1, N08.2, N08.3, N08.4, N08.5, N08.8, N19, Q60, Q60.0, Q60.1, Q60.2, Q60.3, Q60.4, Q60.5, Q60.6, Q61, Q61.0, Q61.1, Q61.2, Q61.3, Q61.4, Q61.5, Q61.8, Q61.9, Q62, Q62.0, Q62.1, Q62.2, Q62.3, Q62.4, Q62.5, Q62.6, Q62.7, Q62.8, Q63, Q63.0, Q63.1, Q63.2, Q63.3, Q63.8, Q63.9, Q64, Q64.0, Q64.1, Q64.2, Q64.3, Q64.4, Q64.5, Q64.6, Q64.7, Q64.8, Q64.9, Z94.0, T80.9, T82.5, T82.7, T82.8, N18.0, N18.8  OR 80.9, T82.5, T82.7, T82.8  AND a disease code for transplant or dialysis: Y60.2, Y61.2, Y62.2, Y84.1, Z49, Z49.0, Z49.1, Z49.2, Z99.2, N16.5, T86.1, Z94.0, N18.5 |
| Nephritic non-hypertensive disease | N11.0, N11.1. N11.8, N11.9, N12 |
| End stage renal disease | N18.5  OR T82.4, Y60.2, Y61.2, Y62.2, Y84.1, Z49, Z49.0, Z49.1, Z49.1, Z99.2, N16.5, T86.1, Z94.0 |
| **Melanoma** |  |
| Squamous cell carcinoma | SDC 1739012 |
| Basal cell carcinoma | SDC 1739003 |

^†^ICD-10 sub-codes will be added where applicable and appropriate.

**Supplementary Table S2. List of dermatologic medications**

| **Medications for treatment of plaque psoriasis or GPP** | |
| --- | --- |
| Topical steroids | ATC code: D07A **OR** hydrocortisone, prednisolone, flumetasone, triamcinolone, fluprednidene, fluorometholone, dexamethasone, betamethasone, desoximetasone, mometasone, diflucortolone, fluocortolone AND route = Topical |
| Other topical medications (non-steroids) | Benzoic acid/salicylic acid, lanolin, nystatin, dimethicone/petrolatum white, dimethicone, aloe vera/petrolatum hydrophilic, miconazole nitrate, tolnaftate, gentian violet, griseofulvin ultramicrosize, clotrimazole, glycerine/mineral oil/petrolatum white/cetostearyl alcohol/water, propylene glycol/mineral oil, ciclopirox olamine, ketoconazole, hydrophilic ointment, terbinafine HCl, sertaconazole nitrate, econazole nitrate, poloxamer/glycerine/dimethicone/water, ciclopirox, undecylenic acid, undecylenic acid/zinc undecylenate, emollient combination no 18, sodium thiosulfate/salicylic acid, cod liver oil/zinc oxide, calcium undecylenate, vitamins/white petrolatum/lanolin, lanolin anhydrous, oxiconazole nitrate, efinaconazole, dimethicone/silicon dioxide, olive oil, tazarotene, tacrolimus, pimecrolimus, coal tar, calcipotriene, calcitriol, anthralin, calcipotriene/betamethasone, dipropionate **OR** ATC code: D05A **AND** route= Topical |
| Systemic steroids (glucocorticoids) | ATC code: H02 **AND** route **not equal to** ‘External Medication’ |
| Tumor necrosis factor inhibitors | Adalimumab (Humira), certolizumab pegol (Cimzia), etanercept (Enbrel), infliximab (Remicade), golimumab (Simponi) |
| Interleukin inhibitors | Ustekinumab (Stelara), secukinumab (Cosentyx), brodalumab (Lumicef), ixekizumab (Taltz), guselkumab (Tremfya), risankizumab (Skyrizi) |
| Any biologic systemic | Adalimumab (Humira), certolizumab pegol (Cimzia), etanercept (Enbrel), infliximab (Remicade), golimumab (Simponi), ustekinumab (Stelara), secukinumab (Cosentyx), brodalumab (Lumicef), ixekizumab (Taltz), guselkumab (Tremfya), abatacept (Orencia), risankizumab (Skyrizi) |
| T-cell inhibitors | Abatacept (Orencia) |
| Phototherapy | Procedure name= Phototherapy |
| Any systemic (biologic or non-biologic), ANY | Adalimumab (Humira), certolizumab pegol (Cimzia), etanercept (Enbrel), infliximab (Remicade), golimumab (Simponi), ustekinumab (Stelara), secukinumab (Cosentyx), brodalumab (Siliq), ixekizumab (Taltz), guselkumab (Tremfya), abatacept (Orencia), risankizumab (Skyrizi)  OR etretinate, apremilast (Otezla), vitamin D3, ciclosporin, hydroxycarbamide, methotrexate, mycophenolate mofetil, salazosulfapyridine, tacrolimus, tofacitinib (Xeljanz), baricitinib (Olumiant), peficitinib (Smyraf), azathioprine  **OR** ATC code: H02 |
| Any systemic (non-biologic, any) | Etretinate, apremilast (Otezla), vitamin D3, ciclosporin, hydroxycarbamide, methotrexate, mycophenolate mofetil, salazosulfapyridine, tacrolimus, tofacitinib (Xeljanz), baricitinib (Olumiant), peficitinib (Smyraf), azathioprine  **OR**  ATC code: H02 |

ATC, Anatomical Therapeutic Chemical Classification; PUVA, psoralen and ultraviolet A.

**Supplementary Table S3. List of medications for comorbidities**

| **Medications for comorbidities** | |
| --- | --- |
| Statins | Atorvastatin, fluvastatin, pravastatin, rosuvastatin, simvastatin, pitavastatin |
| Anti-hypertensives | Angiotensin converting enzyme (ACE) inhibitors, angiotensin receptor blockers, beta-blockers, calcium channel blockers, diuretics, alpha-blockers, alpha-beta-blockers |
| Antibiotics | Penicillins, cephalosporins, macrolides, fluoroquinolones, sulfonamides, tetracyclines, aminoglycosides |
| Type 2 diabetes medication | ATC code: A10 – Drugs used in diabetes |
| Asthma medication | Fluticasone (Arnuity, Flutiform, Flutide), budesonide (Pulmicort), mometasone (Asmanex Twisthaler), beclomethasone (Qvar), ciclesonide (Alvesco), montelukast (Singulair), zafirlukast (Accolate), salmeterol (Serevent), formoterol (Foradil, Perforomist), fluticasone and salmeterol (Adoair), budesonide and formoterol (Symbicort), vilanterol trifenatate/fluticasone furoate (Relvar), fluticasone furoate/umeclidinium bromide/vilanterol trifenatate (Trelegy), salbutamol sulfate, ipratropium (Advent), omalizumab, mepolizumab, benralizumab |
| Chronic obstructive pulmonary disease medication | Salbutamol sulfate, ipratropium, fluticasone, budesonide, aclidinium (Eklira), formoterol, glycopyrronium bromide(Seebri Neohaler), indacaterol, olodaterol, salmeterol (Serevent), tiotropium (Spiriva), umeclidinium (Incruse Ellipta), glycopyrronium/formoterol (Bevespi Aerosphere), glycopyrronium/indacaterol (Utibron Neohaler), tiotropium/olodaterol (Stiolto Respimat), umeclidinium/vilanterol (Anoro Ellipta), budesonide/formoterol (Symbicort), fluticasone/salmeterol (Adoair), fluticasone/vilanterol (Relvar Ellipta) |
| Psychiatric medication | All ATC codes that start with the following:  N05A – Antipsychotics  N05B – Anxiolytics  N05C – Hypnotics and sedatives  N06A – Antidepressants |
| Sleep medication (non-benzodiazepine sedatives) | Eszopiclone, zolpidem |
| Benzodiazepine sedatives | Triazolam, estazolam, lorazepam, flurazepam, quazepam |
| Hyperlipidemia medication | **HMG-CoA reductase inhibitors (statins):** atorvastatin, fluvastatin, pravastatin, rosuvastatin, simvastatin, pitavastatin  **Intestinal cholesterol transporter inhibitor:** ezetimibe  **Resins:** colestimide and cholestyramine  Probucol  **PCSK9 inhibitors:** evolocumab, alirocumab  **MTP inhibitor:** lomitapide  **Fibrates:** bezafibrate, fenofibrate, clinofibrate, clofibrate  **SPPARM alpha:** pemafibrate  **Nicotinic acid derivatives:** niceritrol, nicomol, tocopheryl nicotinate  **PUFAs:** ethyl icosapentate and omega-3-acid-ethyl ester |

ATC, Anatomical Therapeutic Chemical Classification; PUFA, polyunsaturated fatty acid.

**Supplementary Table S4. Added medications of interest**

| **Added medications of interest** | **Search term** |
| --- | --- |
| Acitretin | Etretinate |
| Adalimumab | Adalimumab |
| Apremilast | Apremilast |
| Brodalumab | Brodalumab |
| Certolizumab | Certolizumab pegol |
| Ciclosporine | Cyclosporine, cyclosporine, modified and dosage form = “internal medication” |
| Etanercept | Etanercept |
| Golimumab | Golimumab |
| Guselkumab | Guselkumab |
| Infliximab | Infliximab, infliximab-abda, infliximab-dyyb |
| Ixekizumab | Ixekizumab |
| Methotrexate | Methotrexate, methotrexate sodium, methotrexate sodium/pf, methotrexate/pf |
| Retinoic acid (alitretinoin or isotretinoin) | Tretinoin, tretinoin palmitate |
| Secukinumab | Secukinumab |
| Tofacitinib | Tofacitinib citrate |
| Ustekinumab | Ustekinumab |
| Risankizumab | Risankizumab |
| Vitamin D3 | Vitamin D3 |
| Dovobet | Calcipotriol hydrate + betamethasone dipropionate |
| Maxacalcitol | Maxacalcitol (exclude Marduox |
| Marduox | Betamethasone butyrate propionate |
| Azathioprine | Azathioprine |
| Adacolumn (GMA, apheresis) | Standard material code: 710010609/732030000 |
| Plasma exchange | Plasma exchange |

GMA, granulocyte and monocyte apheresis.
